# Changes in the extracellular microenvironment and osteogenic responses of mesenchymal stem/stromal cells induced by in vitro direct electrical stimulation

**DOI:** 10.1177/2041731420974147

**Published:** 2021-02-16

**Authors:** Kasama Srirussamee, Ruikang Xue, Sahba Mobini, Nigel J Cassidy, Sarah H Cartmell

**Affiliations:** 1 Department of Materials, The University of Manchester, Manchester, UK; 2 Department of Biomedical Engineering, Faculty of Engineering, King Mongkut’s Institute of Technology Ladkrabang (KMITL), Bangkok, Thailand; 3 Instituto de Micro y Nanotecnología IMN-CNM, The Spanish National Research Council (CSIC), Madrid, Comunidad de Madrid, Spain; 4 Departamento de Biología Molecular and Centro de Biología Molecular “Severo Ochoa” (UAM-CSIC), Universidad Autónoma de Madrid, Madrid, Spain; 5 Department of Civil Engineering, University of Birmingham, Birmingham, UK

**Keywords:** Electrical stimulation, mesenchymal stem/stromal cells, computational modelling, faradic by-products, bone regeneration

## Abstract

Electrical stimulation (ES) has potential to be an effective tool for bone injury treatment in clinics. However, the therapeutic mechanism associated with ES is still being discussed. This study aims to investigate the initial mechanism of action by characterising the physical and chemical changes in the extracellular environment during ES and correlate them with the responses of mesenchymal stem/stromal cells (MSCs). Computational modelling was used to estimate the electrical potentials relative to the cathode and the current density across the cell monolayer. We showed expression of phosphorylated ERK1/2, c-FOS, c-JUN, and SPP1 mRNAs, as well as the increased metabolic activities of MSCs at different time points. Moreover, the average of 2.5 μM of H_2_O_2_ and 34 μg/L of dissolved Pt were measured from the electrically stimulated media (ES media), which also corresponded with the increases in SPP1 mRNA expression and cell metabolic activities. The addition of sodium pyruvate to the ES media as an antioxidant did not alter the SPP1 mRNA expression, but eliminated an increase in cell metabolic activities induced by ES media treatment. These findings suggest that H_2_O_2_ was influencing cell metabolic activity, whereas SPP1 mRNA expression was regulated by other faradic by-products. This study reveals how different electrical stimulation regime alters cellular regenerative responses and the roles of faradic by-products, that might be used as a physical tool to guide and control cell behaviour.

## Introduction

Following the observation of bone regeneration around the direct current (DC) cathode by Yasuda^1^ in 1953, there have been a number of the pre-clinical studies on the applications of electrical stimulation (ES) in orthopaedics reported in the literature.^2–9^ The idea of using ES in modern orthopaedics has since become attractive, and in 1979, the use of ES was approved by the FDA for non-union fracture treatments.^10,11^ Currently, there are more than 10 types of commercial ES devices available from companies such as Zimmer Biomet and Orthofix.^12,13^

In general, there are several methods for delivering ES to bone tissue, including direct methods (e.g. implanted electrodes), and indirect methods (e.g. capacitive coupling and inductive stimulation).^14,15^ Pre-clinical and clinical studies showed that all of these methods were capable of promoting bone healing to some extent, particularly in spinal fusion.^11–13^ The stimulating effects of ES could be transmitted to the cells in the form of an electrostatic field, electromagnetic field, or electric current, depending on the configuration of the ES device.^14,15^ For example, the capacitive coupling delivers the electrostatic field to the tissue, whereas the inductive ES delivers the electromagnetic field.^14,15^ On the other hand, direct ES with the implanted electrodes delivers the electric current through the interfaces between the electrode and the tissue.^14–16^ The current delivered by direct ES includes non-faradic and faradic charges. Non-faradic charge is delivered through the re-distribution and adsorption of the charged ions at the electrode surfaces to form the double layer, whilst faradic current is a result of charge injection into electrolyte that is responsible for the electrochemical reactions.^16–18^ However, the exact underlying cellular and molecular mechanisms that regulated the electrically induced cell mineralisation in vitro and bone deposition in vivo were not completely elucidated, and thus this study aimed to explore further to understand the mechanism of action and the potential stimulating factors of direct ES.^11,13^

According to the literature, it appears that every method of ES altered the intracellular calcium level by inducing either calcium influx or calcium release from intracellular storage, which promoted osteoblast proliferation and osteogenic protein expressions from mesenchymal stem/stromal cells (MSCs).^19–21^ This could be due to the accumulation of charges on cell membrane that eventually led to the opening of the voltage gated calcium channels.^22^ Moreover, changes in intracellular calcium level as a result of ES could also influence cell migration and orientation via electrotaxis.^21,23–28^ Calcium-dependent responses to ES could also influence the expressions of bone morphogenetic proteins (BMPs), collagen type I, and other cellular osteogenic activities.^29–39^ In addition to calcium, the distribution of other soluble ions and their channels on the cell membrane as well as the molecules, which play a key role in cell function, such as sodium, potassium, adenosine triphosphate (ATP), and reactive oxygen species (ROS), were also tailorable by ES.^40–46^

It is noted that not only delivery method of ES that could cause variation in cellular responses and the clinical outcome, but also the electrode systems and materials. Direct ES method, in particular, is the only method that involved direct contact between electrode and tissue.^14,15^ In the in vitro models, various electrodes were used, such as carbon, metals, conductive polymers, and salt bridges.^47^ However, it appears that the majority of the implantable orthopaedic ES devices used clinically and those used in vivo were composed of bare metallic electrodes, such as titanium, platinum, and stainless steel.^1–4,48–50^ One of the key characteristics of bare electrode was the generation of faradic by-products from electrochemical reactions.^9,14,15,46^ These reactions were not applicable in the salt bridge system, since the salt bridges have isolated the cells from electrode contact and the ionic current was delivered without the by-products.^15,47^ The by-products from cathodic reactions, such as the hydroxyl ions and reactive oxygen species (ROS) generated by oxygen reduction reaction, were particularly of interests based on the early observation of new bone formation around the cathode in vivo.^1–4,48–51^ These by-products have been hypothesised to be involved in the mechanism of electrically induced osteogenesis.^9,11,51–53^ The suggested mechanisms include the enhancement of osteoblastic activities and the suppression of osteoclastic activities by an increase in pH.^11,53–55^ On the other hand, the electrically generated H_2_O_2_ was suggested to trigger osteoclastic bone resorption and VEGF secretion from macrophages, which could promote bone remodelling and vascularisation.^11,51,53,56,57^ Moreover, the recent study has found that the faradic by-products could also influence the SPP1 mRNA expression from macrophages.^34^ This response was relevant to osteogenesis because SPP1, or osteopontin, is one of the bone matrix proteins and is also a biomarker for the osteogenic activities of bone cells.^34^

It has been reported that ROS also played a role in initiating cellular signalling and activities of the MSCs.^58–61^ This could be beneficial for bone repair and regeneration as MSCs were capable of differentiating towards the osteoblastic phenotypes in vitro and secreting the bioactive factors in vivo.^62–65^ Hence, in this study, we investigated and analysed the initial mechanism of action of direct ES and the role of faradic by-products, in particular. It was hypothesised that the MSC activities could be regulated by the faradic by-products during the application of direct ES. This study addressed this hypothesis by using end-to-end experimental design, starting from characterising the changes in extracellular microenvironment induced by ES towards the evaluation of the responses from human bone marrow-derived MSCs (hBM-MSCs), including protein expression, mRNA expression, and cell proliferation. Computational modelling was used to visualise the changes in the electrolyte potential and current density during direct ES, and the generation of faradic by-products in the culture media was also characterised. These changes were discussed in relation to the observed cellular responses in order to deconstruct the potential stimulating factors during direct ES.

## Materials and methods

### Cell culture and culture media preparation

Commercial primary human bone marrow-derived MSCs (hBM-MSCs) (Lot No. 6F4392, Lonza) were expanded in MSC Growth Medium 2 (PromoCell) containing 1% antibiotic antimycotic solution. Cells were cultured at 37°C, 5% CO_2_. All reagents used in this study were supplied from Sigma-Aldrich, UK, unless stated otherwise. In the experiments, Dulbecco’s modified Eagle’s medium (DMEM) containing 4.5 g/L glucose, 4 mM L-glutamine, and without sodium pyruvate was used. DMEM was supplemented with 10% heat inactivated foetal bovine serum (FBS) and 1% antibiotic antimycotic solution before use as growth media (GM). 100 mM sodium pyruvate solution was further diluted into GM as required. Cells at passage 7 were seeded into six-well plates at the density of 10,000 cells/cm^2^ with 3 ml of GM. Media were changed the day after seeding accordingly to the experimental condition and every 3 days afterwards. Osteogenic medium (OM) was prepared by supplementing GM with 10 mM β-glycerophosphate, 0.17 mM (50 µg/ml) L-ascorbic acid 2-phosphate, and 10 nM dexamethasone. Cells were cultured until reaching the time point when the data were collected.

### Direct ES system

**Figure 1.**
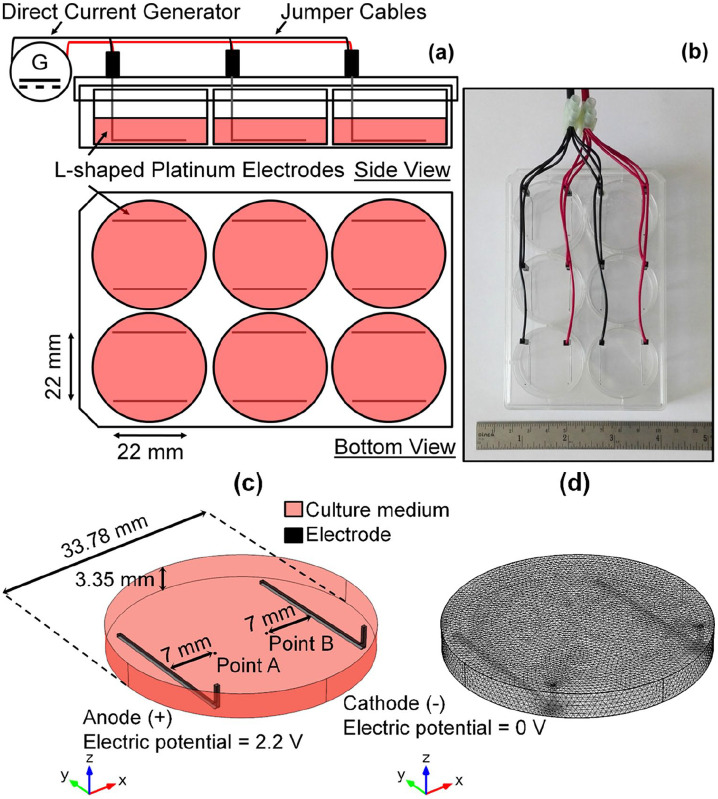
Direct electrical stimulation device used in this study. (a) Schematic diagram and (b) representative image of the assembled devices. (c) Computer-aided geometry and (d) meshed structure used in computational modelling.

The ES chambers used in this study were adapted and modified from the original design by Mobini and Leppik, et al.^37,38^ L-shaped platinum (Pt) electrodes (99.95% platinum wires with 0.5 mm diameter) were fitted into standard 6-well plate lid as shown in Figure 1(a) and (b), similarly to those used in our previous study.^34^ Constant direct current (DC) voltage of 2.2 V was supplied from the generator (B&K Precision). This regime was not invasive for when applied directly to the MSCs in vitro (direct ES) for 1 h daily, and thus it was used throughout the study.^38,39,49^ In direct ES experiments, the control groups were non-stimulated cells cultured in GM and OM. In the case of electrically stimulated media (ES Media), acellular media were incubated overnight before being exposed to ES for 1 h. The ES media, which contain faradic by-products, were transferred to the cells immediately after stimulation every day to mimic daily ES.^34^ In the ES media experiments, the control groups were cells treated with non-stimulated media (Control Media) with and without sodium pyruvate.

### Computational electrochemistry modelling and validation

A computational model was built with COMSOL^®^ Multiphysics 5.2 (COMSOL AB). *Secondary Current Distribution* interface from *Electrochemistry* module was used. This module calculated the transport of charged ions in an electrolyte and current conduction in electrodes based on Ohm’s law with a charge balance:

(1) i=−σ∇ϕ

(2) ∇⋅i=Q

where *i* is the current density vector (A/m^2^), *σ* is the conductivity (S/m), *ϕ* is the potential (V), and *Q* is the general current source term (A/m^3^).

Furthermore, it also took into account the activation overpotentials due to charge transfer reactions, described by the Butlet-Volmer equation:

(3) $$i=i_{0}\left(\exp\left(\frac{\alpha_{a}F\eta }{RT}\right)-\exp\left(\frac{-\alpha_{c}F\eta }{RT}\right)\right)$$

where *i* is the electrode current density (A/m^2^), *i_0_* is the exchange current density (A/m^2^), *α_a_* is the anodic transfer coefficient, *α_c_* is the cathodic transfer coefficient, *F* is the Faraday’s constant (C/mol), *η* is the activation overpotential (V), *R* is the gas constant (J/(K mol)), and *T* is the temperature (K). The activation overpotential was defined in the following way:

(4) $$\eta =E_{electrode}-E_{eq}$$

where *E_electrode_* is the electrode potential and *E_eq_* is the equilibrium potential for the electrode reaction.

Figure 1(c) shows the computer-aided model that was composed of 3-ml culture medium and two L-shaped Pt electrodes. The electrodes were placed 22 mm apart in parallel. In the model assumption, the anode underwent oxidation reaction, whilst the cathode underwent reduction reaction. Cell voltage between 0 and 5 V was applied at the anode, and ground voltage was applied at the cathode. Assuming symmetric electrochemical reactions, the equilibrium potential magnitude at each electrode were equal. The parameters used in the simulation are tabulated in Table 1. 207,982 tetrahedral elements were generated with the mesh size ranging from 0.00676 to 0.676 mm, as shown in Figure 1(d). The electrolyte potential relative to the cathode at arbitrary points: A; and B, at each applied voltage were simulated and compared against the experimental values for model validation. The electrolyte potential and current density distribution across the *x*-*y* and *y*-*z* surfaces under the similar direct ES condition to those used for cell culture experiments were simulated and visualised.

**Table 1.** Parameters used in the computational model.

Name	Expression	Description	Reference
alpha_a	0.5	Anodic transfer coefficient	Kumsa et al.^66^
alpha_c	0.5	Cathodic transfer coefficient	
Ecell	0–5 V	Applied voltage	n/a
Eeq_a	$\frac{E_{\mathrm{cell}}}{2}\left(\mathrm{at}\;E_{\mathrm{cell}}\leq 1.5V\right)$	Anode equilibrium potential	Empirical data (supplemental information)
	$\frac{E_{\mathrm{cell}}}{2}\left(1-\left(0.0000343e^{\left(2.564E_{cell}\right)}\right)\right)$ $\left(\mathrm{at}\;1.5\;V<E_{\mathrm{cell}}<3.0\;V\right)$		
	$\frac{E_{\mathrm{cell}}}{2}\left(1.3558-0.1444E_{cell}\right)\left(\mathrm{at}\;E_{\mathrm{cell}}\geq 3.0\;V\right)$		
Eeq_c	$-\frac{E_{\mathrm{cell}}}{2}\left(\mathrm{at}\;E_{\mathrm{cell}}\leq 1.5V\right)$	Cathode equilibrium potential	
	$-\frac{E_{\mathrm{cell}}}{2}\left(1-\left(0.0000343e^{\left(2.564E_{cell}\right)}\right)\right)$		
	$\left(\mathrm{at}\;1.5\;V<E_{\mathrm{cell}}<3.0\;V\right)$		
	$-\frac{E_{\mathrm{cell}}}{2}\left(1.3558-0.1444E_{cell}\right)\left(\mathrm{at}\;E_{\mathrm{cell}}\geq 3.0V\right)$		
i0_a	4 A/m^2^	Anode exchange current density	Kita^67^
i0_c	4 A/m^2^	Cathode exchange current density	
T	298 K	Temperature	n/a
Sigma	1.5 S/m	Electrolyte conductivity	Mittal et al.^68^

Note: The **bold** coefficients from empirically fitted equations are dimensionless.

For total current measurement, digital multimetre (M-830B, Sinometer) was connected in series to one representative chamber of the ES device, and the other chambers were disconnected from the circuit. Data were recorded at the applied voltage between 0 and 5 V. For electrolyte potential measurement, ground of the multimetre was connected to the ground channel of the generator, whilst the Pt wire was used as a measuring probe. The probe was submerged into the culture media through the guided holes on the device during direct ES to measure the potential at point A and B relative to the cathode. Each point was located on the diameter of the well and 7 mm away from the anode and the cathode, respectively. The measurements were conducted at room temperature (RT).

### H_2_O_2_ concentration measurement

Fluorometric hydrogen peroxide assay kit (MAK165, Sigma-Aldrich) was used to measure H_2_O_2_ concentration. It comprises assay buffer, 20 U/ml horseradish peroxidase, and red peroxidase substrate. Working solution was prepared based on manufacturer’s instructions. 50 µl of samples were transferred into 96-well plate followed by the addition of 50 µl working solution and 30-minute RT incubation, respectively. Fluorescence intensity was measured at excitation wavelength of 544 nm and emission wavelength of 590 nm. The measured intensity was subtracted by background readings. Concentration of H_2_O_2_ was calculated using the calibration curve from standard H_2_O_2_ solution supplied with the kit diluted in GM. In the experiment, acellular media were incubated overnight prior to the experiment, and the measurement was carried out immediately after stimulation.

### Inductively coupled plasma mass spectroscopy

Serum-free acellular GM samples were incubated overnight before applying ES for 1 h. The stimulated samples were filtered through 0.45 μm membrane and diluted 50 times in 2% of 1:1 HNO_3_ and HCl mixture before being analysed for the dissolved platinum content using Inductively coupled plasma mass spectroscopy (ICP-MS) spectrometer (7700×, Agilent).

### Fluorescence staining of ERK1/2

Cells were cultured on glass coverslips placed in 6-well plate treated with GM. Immediately after 30 min and 1 h of ES, cell monolayers were washed with Dulbecco’s phosphate-buffered saline (DPBS), fixed with 10% neutral buffered formalin solution for 10 min, and washed three times with DPBS. The fixed samples were permeabilised by 0.2% Triton^®^ X-100 in DPBS for 10 min and washed three times with DPBS containing 1% bovine serum albumin (BSA) and 0.1% Tween^®^ 20 (VWR), hereafter denoted by PBST. The permeabilised samples were incubated at RT for 30 min with 10% goat serum in PBST and stained with rabbit anti-phosphorylated ERK1/2 (T202/T185) (Abcam) diluted 1:100 in PBST at 4°C overnight. The samples were washed again with PBST three times and stained with goat anti-rabbit Alexa Fluor^®^ 488 (Abcam) diluted 1:1000 in PBST and 5 mg/ml 4′,6-diamidino-2-phenylindole (DAPI) (Thermo Fisher Scientific) diluted 1:5000 in PBST at room temperature for 1 h. Finally, the samples were mounted on the microscope slides using Fluoroshield mounting medium (Abcam). Images were captured using a confocal laser scanning microscope (TCS SP5, Leica) at excitation/emission wavelength of 488/520 and 405/455 nm.

### mRNA extraction and real-time quantitative reverse transcription PCR (RT-qPCR)

mRNA was extracted from the samples after 1 h and after 7 days of direct ES or ES media treatment using spin columns (RNeasy Mini Kit, Qiagen) following the supplier’s protocol. Briefly, cell monolayers were washed two times with DPBS before being scraped off and homogenised in the supplied lysis buffer RLT using syringes and 25G needles. Seventy percent ethanol was added into the lysates before passing through the spin columns via centrifugation. The columns were then washed with supplied washing buffers: RW1; and RPE, and the purified mRNAs were eluted by passing RNase-free water through the columns. Final mRNA concentration was measured using NanoDrop™ Lite spectrophotometer (Thermo Fisher Scientific).

RT-qPCR was carried out using QuantiFast SYBR^®^ Green RT-PCR Kit and QuantiTect Primer Assay (Qiagen). Details of the primers are shown in Table 2; however, their sequences are proprietary. Final reaction volume of 25 μl was composed of 15.25 µl of reaction mix and 9.75 µl of mRNA sample (9 ng). Reverse transcription was carried out at 50°C for 10 min. PCR was activated at 95°C for 5 min before entering cycle stage, which consisted of 10-s denaturation at 95°C and 30-s annealing/extension at 60°C. Fluorescence data were recorded up to 40 cycles using StepOnePlus™ Real-Time PCR System and StepOnePlus™ Software v2.3 (Thermo Fisher Scientific). Fold expression was calculated using ΔΔC_T_ method in relative to the endogenous reference gene (GAPDH) and normalised to the samples from Control (non-stimulated) group.

**Table 2.** Details of the primers used in this study.

Target gene	Assay ID	Translated protein
Glyceraldehyde-3-phosphate dehydrogenase (GAPDH)	Hs_GAPDH_1_SG	GAPDH
c-FOS	Hs_FOS_1_SG	c-FOS
c-JUN	Hs_JUN_1_SG	c-JUN
Runt-related transcription factor 2 (RUNX2)	Hs_RUNX2_1_SG	RUNX2
Secreted phosphoprotein 1 (SPP1)	Hs_SPP1_1_SG	Secreted phosphoprotein 1 or osteopontin

### Cell metabolic activity assay

Resazurin assay (Deep Blue Cell Viability™ Kit, BioLegend) was used in this study to determine cell metabolic activity after 10 days of direct ES or ES media treatment. The stock solution was diluted in GM to make 10% working solution. To implement the assay, cell culture supernatant was replaced with 1 ml of working solution before being incubated at 37°C for 1 h. Fluorescence intensity was measurement at excitation wavelength of 544 nm and emission wavelength of 590 nm. The measured intensity was subtracted by background readings.

### Alkaline phosphatase (ALP) activity assay

ALP activity was quantified after 10 days of direct ES using commercial colorimetric assay kit (K412, BioVision). Cell monolayers were washed two times with DPBS before being scraped off and homogenised in 500 µl assay buffer by passing through 25G needles. Cell lysates were centrifuged and 20 µl of supernatants were used in the assay. About 50 µl of 5 mM p-nitrophenyl phosphate (pNPP) was added to the samples followed by 1 h incubation at room temperature. Stop solution was used to inhibit the reactions in negative control samples and also to terminate the reactions of the samples after incubation. Absorbance of the mixtures was measured at the wavelength of 412 nm and subtracted by the absorbance of negative control samples. Data were normalised to cell metabolic activity measured from resazurin assay.

### Statistical analysis

Data were collected from between two and six samples (replicates) per experiment, which were stimulated by different pairs of electrodes. There were three experiments conducted in parallel, and different ES devices were used for each experiment. Parametric tests were used in this study due to small sample size, and the testing methods are described in the figure legends. The analyses were carried using GraphPad Prism 7 software (GraphPad Software, USA). *p*-values of less than 0.05 were considered statistically significant.

## Results

### Computational electrochemical modelling and validation

**Figure 2.**
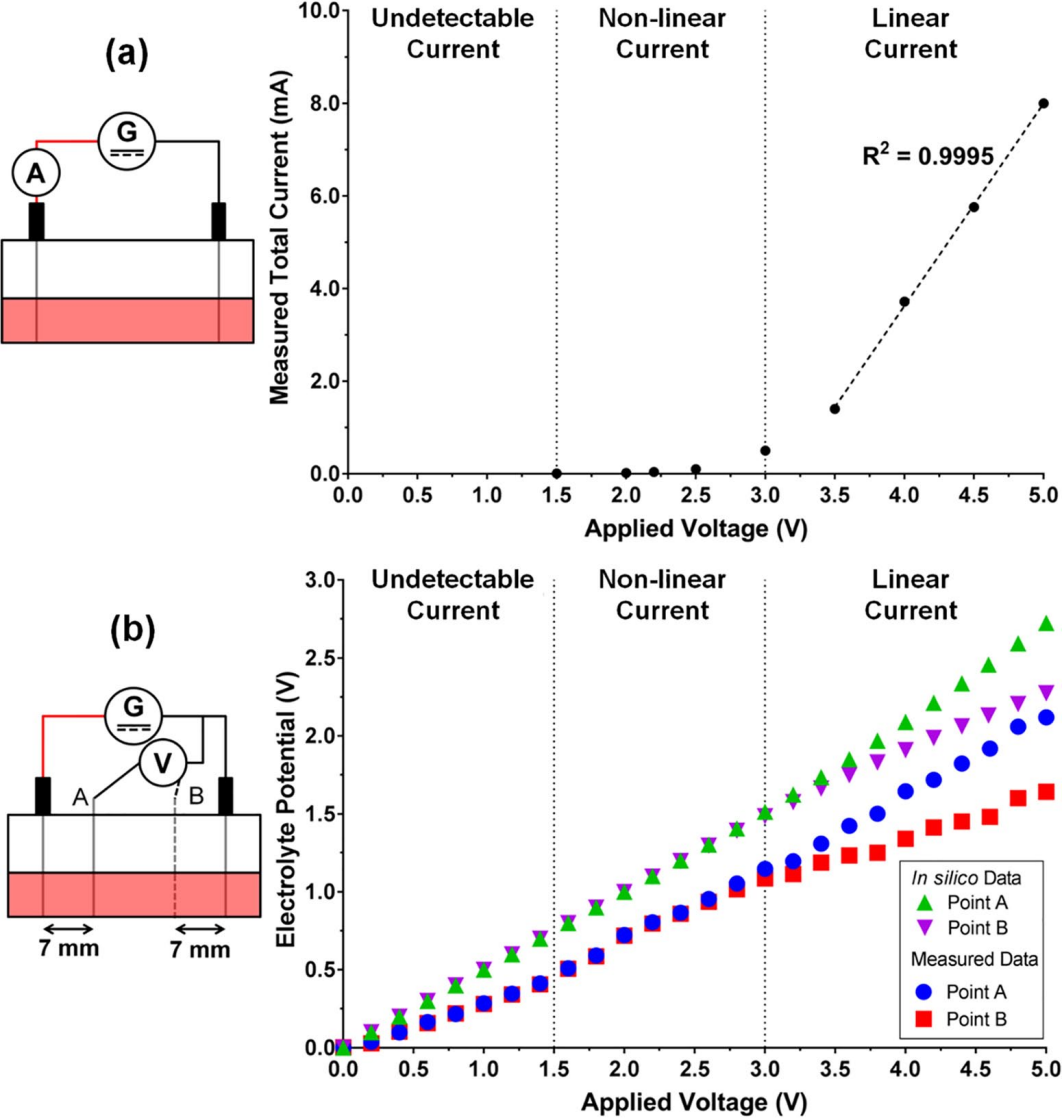
(a) Measured total current passing through one representative chamber during electrical stimulation (ES). (b) Computational (*in silico*) and measured electrolytic potential in one representative chamber during ES. The associated front-view schematic diagrams of each experimental set-up are shown on the left.

The total current passing through the well during ES at the applied voltage of up to 5 V was measured in order to determine the empirical coefficients for the equilibrium potentials at the electrodes as described in Supplemental information and Figure S1. Figure 2(a) shows the measured total current in function of the applied voltage, in which the relationship was non-linear between 1.5 and 3.0 V and was linear from round 3.5 V and higher. Furthermore, the computed electrolyte potentials relative to the cathode using the obtained empirical coefficients are shown in Figure 2(b) together with the measured potentials. It was found that the magnitude of the measured electrolyte potentials was smaller than those obtained from the computational modelling, although both datasets are consistent in terms of their trends.

**Figure 3.**
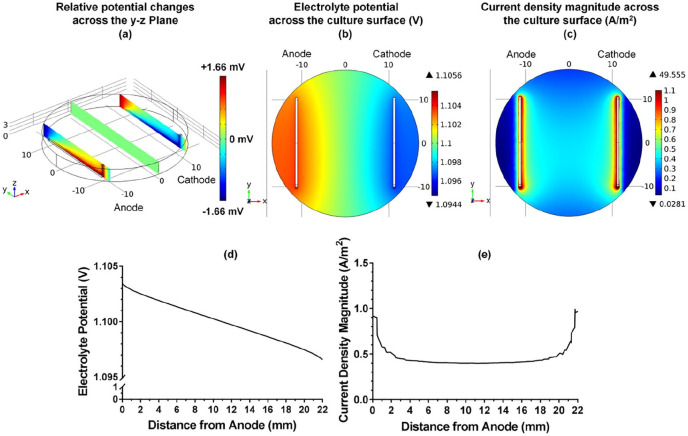
(a and b) Distribution of the computational electrolyte potential relative to the cathode at the applied voltage of 2.2 V across the *y*-*z* plane and across the *x*-*y* plane (cell culture surface), respectively. (c) Computational current density magnitude distribution across the *x*-*y* plane at the applied voltage of 2.2 V. Scale grids are in mm. (d and e) Computational electrolyte potential and current density magnitude between two electrodes at the centre of the well at the applied voltage of 2.2 V.

The distribution of electrolyte potential and current density magnitude in the chamber were further visualised at the applied voltage of 2.2 V, which represented the condition used in cell culture experiment. It was found that the distribution along *z* direction was dependent on the shape of electrodes, whilst the distribution in the middle of the chamber was homogenous, Figure 3(a). Furthermore, slight heterogeneity of the potential distribution across the cell culture surface could also be noticed, and the potential theoretically sensed by the cells during direct ES was in the range between 1.09 and 1.12 V relative to the cathode, Figure 3(b) and (d). On the other hand, the current density magnitude was much higher in the vicinity of the electrodes than in the middle area, as shown in Figure 3(c). The maximum current density magnitude was observed at the electrode surfaces, which dramatically decreased within 3-mm distance from the anode and cathode before reaching plateau of approximately 0.5 A/m^2^ at the centre of the well, Figure 3(e).

### Acellular media characterisation

**Figure 4.**
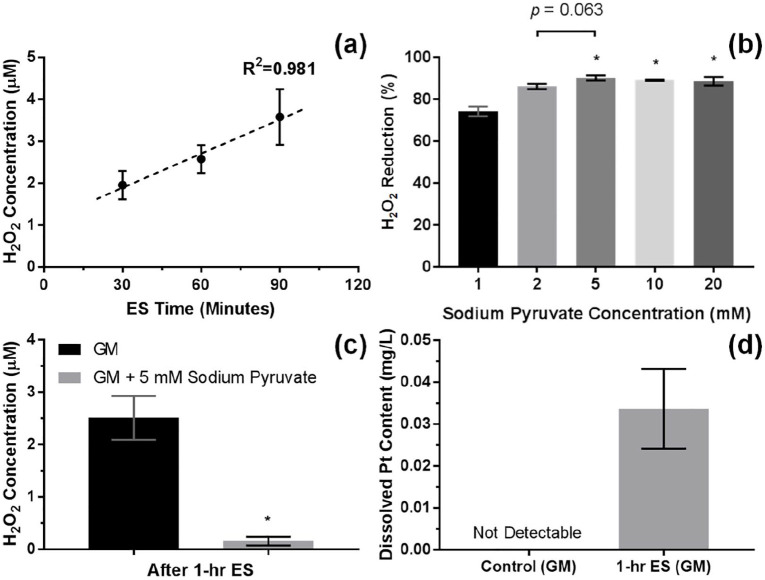
(a) H_2_O_2_ concentration of the acellular growth media (GM) in function of the electrical stimulation (ES) time. Error bars represent SD (*n* = 6 replicates), and dash line represents linear trend line. (b) The reduction in H_2_O_2_ concentration of the acellular GM after 1 h of ES by sodium pyruvate ranging from 1 to 20 mM relative to 0 mM. Error bars represent SD (*n* = 3 replicates). * represents *p* < 0.05 (one-way ANOVA with Tukey’s pairwise comparison) when compared with 1 mM. (c) H_2_O_2_ concentration of the acellular GM after 1 h of ES without and with 5 mM sodium pyruvate. Error bars represent SD (*n* = 3 experiments). *represents *p* < 0.05 (unpaired two-tailed Student’s *t*-test) when compared with GM without sodium pyruvate. (d) Measured dissolved Pt content in the acellular GM after 1 h of ES using ICP-MS. Error bars represent SD (*n* = 3 experiments).

The results showed that the average H_2_O_2_ concentration in the acellular media was increased to 2.5 μM after 1 h of stimulation, and changes in H_2_O_2_ concentration were linearly proportional to the stimulation time within 30–90 min range, as shown in Figure 4(a). Moreover, the performance of sodium pyruvate as an antioxidant was also tested. It was seen that the H_2_O_2_ concentration increased by 1-h ES was reduced by around 90% with sodium pyruvate supplementation at 5 mM or higher, Figure 4(b) and (c). The elemental analysis from ICP-MS has shown a significant increase in dissolved Pt content in the culture media to around 34 µg/L after 1-h ES, Figure 4(d).

### Phosphorylated ERK1/2 staining

**Figure 5.**
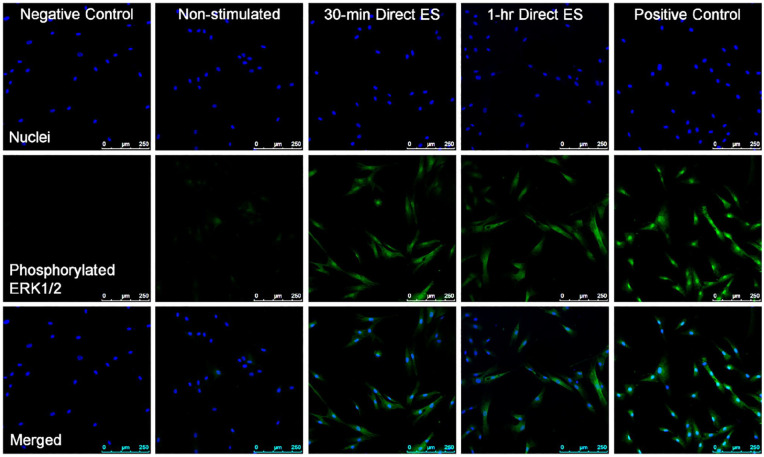
Fluorescence images of cells after 30 min and 1 h of electrical stimulation in comparison with non-stimulated cells with 10× optical magnification. Nuclei are stained blue, and phosphorylated ERK1/2 is stained green. Scale bars = 250 µm. The images are representative from three experiments. Positive control samples are cells treated with 200 ng/ml phorbol 12-myristate 13-acetate (PMA) for 30 min, and negative control samples are also PMA-treated cells with the absence of primary antibody staining.

Figure 5 shows the fluorescence images indicative of phosphorylated ERK1/2 from cells electrically stimulated for 30 min and 1 h in comparison with non-stimulated cells. It was noticeable that direct ES has activated the phosphorylation of ERK1/2 within the first 30 min of stimulation, and the increased expression of phosphorylated ERK1/2 was still detectable after 1 h of direct ES.

### mRNA expressions and cell metabolic activity

**Figure 6.**
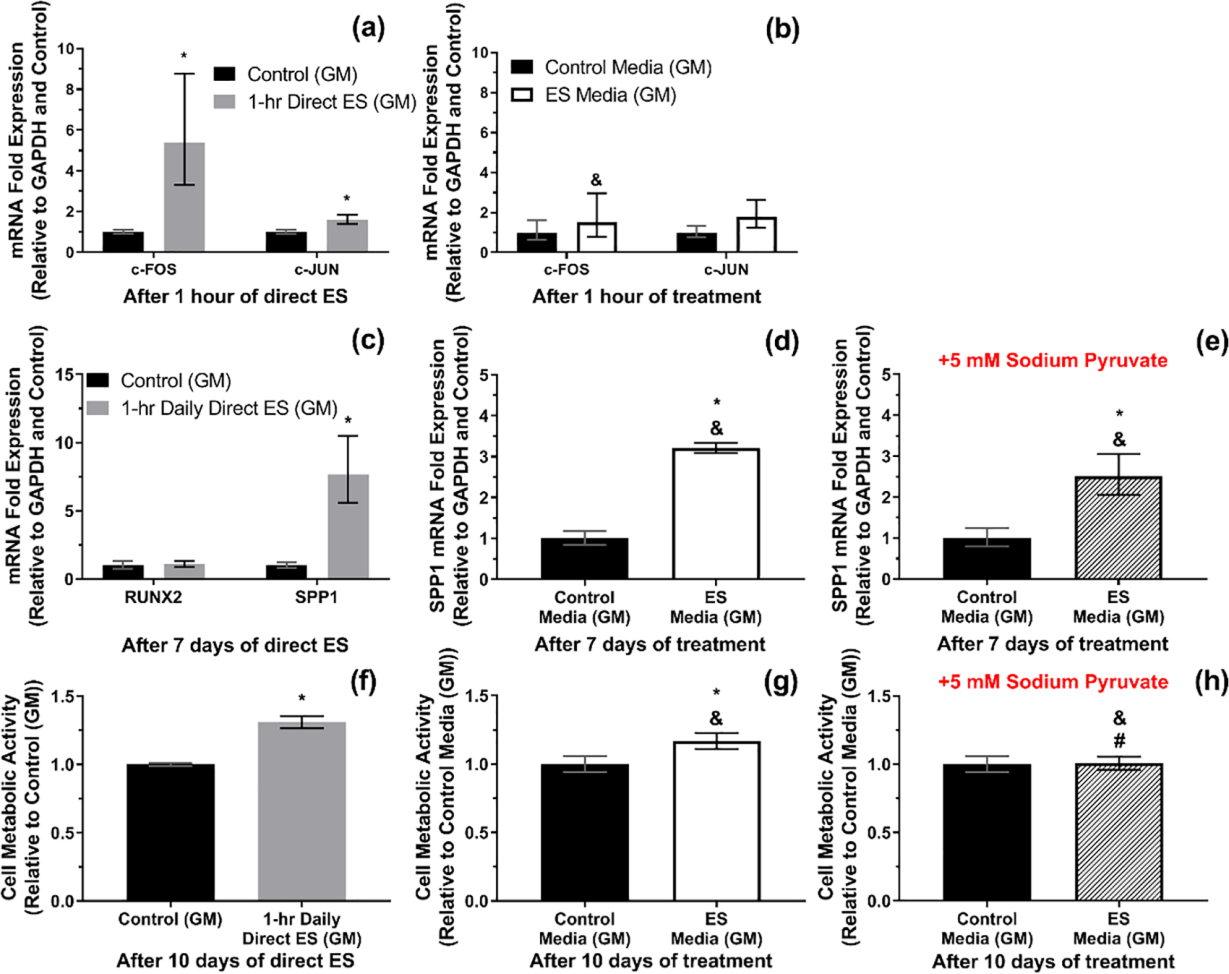
(a and b) c-FOS and c-JUN mRNA expressions after 1 h of direct electrical stimulation (ES) and after 1 h of exposure to the electrically stimulated media (ES media), respectively. (c) RUNX2 and SPP1 mRNA expression after 7 days of 1-h daily direct ES. (d and e) SPP1 expression after 7 days of treatment with ES media and ES media containing 5 mM sodium pyruvate, respectively. (f–h) Cell metabolic activity after 10 days of 1-h daily direct ES, treatment with ES media, and treatment with ES media containing 5 mM sodium pyruvate, respectively. Error bars in (a–e) represent upper and lower 95% confidence limits (*n* = 3 experiments). Error bars in (f–h) represent SD (*n* = 3 experiments). * represents *p* < 0.05 (unpaired two-tailed Student’s *t*-test) when compared with Control (non-stimulated) group. In (b), & represents *p* < 0.05 (unpaired two-tailed Student’s *t*-test) when compared with Direct ES group. In (d, e, g and h), & and # represent *p* < 0.05 (one-way ANOVA with Tukey’s pairwise comparison) when compared with Direct ES and ES media without sodium pyruvate groups, respectively.

Figure 6(a) shows that the expression of c-FOS and c-JUN mRNAs were significantly upregulated after 1 h of direct ES. However, these were not the case when treating the cells with the faradic by-products from the electrically stimulated media (ES media) for 1 h (*p* > 0.05), Figure 6(b). Furthermore, after 7 days of 1-h daily direct ES, a significant upregulation of SPP1 mRNA expression has been observed, whereas the level of RUNX2 mRNA expression was not affected by ES, Figure 6(c). Changes in the expression of c-FOS and c-JUN mRNAs were no longer significant after 7 days of 1-h daily direct ES, supplemental Figure S2. An increase in SPP1 expression was also observed when treating the cells with ES media for 7 days, and the supplementation of 5 mM sodium pyruvate did not alter the level of SPP1 expression induced by ES media, Figure 6(d) and (e).

On the other hand, it was found that cell metabolic activity was significantly increased after 10 days of 1-h daily direct ES, and this trend was also found when treating the cells with ES media for 10 days, as shown in Figure 6(f) and (g). However, the supplementation of 5 mM sodium pyruvate has suppressed the effect of ES media on cell metabolic activity, Figure 6(h). It was noted that the level of c-FOS and SPP1 mRNA expressions and cell metabolic activity as a result of direct ES were significantly different from the responses from cells treated with ES media with and without sodium pyruvate.

### mRNA expressions, cell metabolic activity, and ALP activities in osteogenic media (OM)

**Figure 7.**
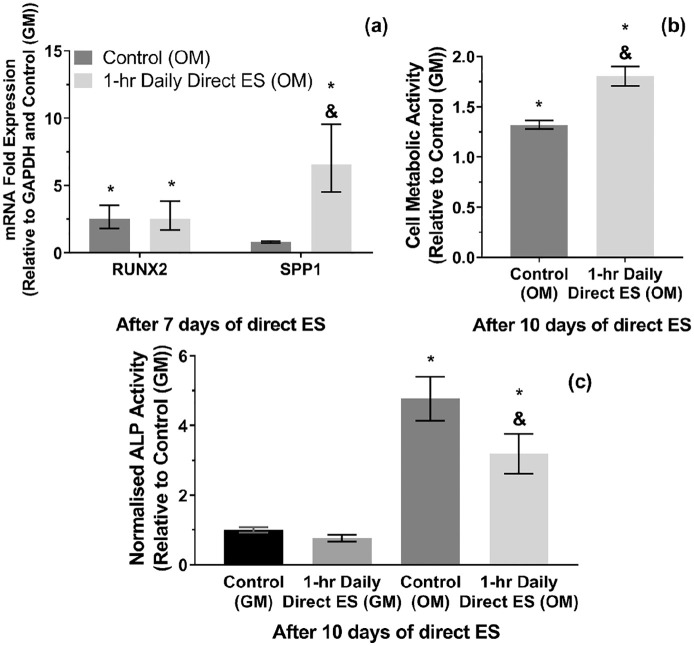
(a) RUNX2 and SPP1 m RNA expressions after 7 days of 1-h daily direct ES in osteogenic media (OM). (b) Cell metabolic activity and (c) ALP activity after 10 days of 1-h daily direct ES in OM. Error bars in (a) represent upper and lower 95% confidence limits (*n* = 3 experiments). Error bars in (b and c) represent SD (*n* = 3 experiments). * and & represent *p* < 0.05 (two-way ANOVA with Tukey’s pairwise comparison) when compared with Control (GM) and Control (OM) groups, respectively.

It could be seen in Figure 7(a) that the upregulation of SPP1 mRNA expression as a result of direct ES was also detectable when culturing the cells in OM. Although RUNX2 mRNA expression has increased by around 2.5 folds when treating the cells with OM in non-stimulated group, there was still no significant difference between stimulated and non-stimulated cells in OM. Likewise, an increase in cell metabolic activity after direct ES was also significant in OM-treated cells, as shown in Figure 7(b). Moreover, OM treatment has significantly increased the cellular ALP activity after 10 days, although it appeared that the stimulated cells exhibited lower level of ALP activity than non-stimulated cells, Figure 7(c).

## Discussion

This study shows that direct ES has changed the physical and chemical conditions of the extracellular microenvironment, which consequently influenced the activities of MSCs. The effects of each stimulating factors were evaluated by comparing the cellular responses to direct ES and to the medium that was conditioned by ES (ES media) in presence or absence of sodium pyruvate as an antioxidant, in which the effect of electrical factors, electrically generated H_2_O_2_, and other types of faradic by-products could be discussed. Interestingly, our results showed that changes in MSCs responses were influenced by both faradic and non-faradic charges, which supported the hypothesis of this study that the MSC activities could be regulated by the faradic by-products. Although we have shown previously that faradic by-products could influence the activities of macrophages, here we suggest that MSCs also respond to the faradic by-products that could contribute to the mechanism of electrically induced osteogenesis.^34^

### Changes in microenvironment under direct ES and faradic by-products generation

Computational modelling has been an effective tool for voltage optimisation and the visualisation of electric field during ES in human bone and skin tissues as well as in the in vitro model.^69–71^ In this study we utilised similar technique to visualise and calculate the physical changes in microenvironment under in vitro direct ES. The results suggested that the voltage applied directly to the culture media should not exceed 3.0 V to avoid water splitting and excessive electrolysis that are cytotoxic. Moreover, we showed that, in our electrical stimulation system, the electrolyte potential increased by direct ES exhibited the voltage loss of approximately 30% from the computational values, which was observable in the system that involves charge transfer across the electrode-electrolyte interfaces.^72^ The computed current density magnitude at the centre of the chamber during direct ES in our study was less than the threshold of 5 A/m^2^ that could cause cell or tissue necrosis.^9,14^ However, it should be noted that cell necrosis was still expectable at the area close to the electrode surfaces and previously was found to be significant in pre-osteoblasts and macrophages.^34^ In terms of the computational model, it is understood that the model used in this study still requires an improvement for its boundary conditions as it currently relies on the empirical input and did not cover the effect of Helmholtz double layer at the electrode-electrolyte interfaces.

Apart from these physical changes, this study also investigated the chemical changes in culture media as a result of ES. It was reported earlier that the direct ES could generate OH^-^ and H_2_O_2_ as well as trace elements from electrode materials as a result of electrochemical reactions.^51,73^ Moreover, this particular direct ES system and regime was further reported in our previous study that pH of the culture media did not significantly change, whereas the electrically generated H_2_O_2_ was detectable after 1 h of ES.^34^ It was also found in this study that the electrically generated H_2_O_2_ concentration was linearly proportional to the stimulation time within 30 to 90 min range. On the other hand, the trace of Pt, which was our electrode material, was detectable at around 34 µg/L in the electrically stimulated media. This level of Pt dissolution was lower than the cytotoxic concentration reported previously that the viability of murine fibroblasts and human neuroblastoma were significantly reduced at the Pt dissolution concentration of 1.64 mg/L or higher.^74^ Consistently, the cytotoxic effects of Pt dissolution in this study were not statistically significant. Furthermore, it was found that sodium pyruvate could neutralise the electrochemically generated H_2_O_2_ from this direct ES system by converting H_2_O_2_ into acetate, CO_2_, and water.^75^ The aforementioned changes in the culture media were the identified faradic by-products in this study. In addition, it is worth noting that the generation of faradic by-products was also dependent on chemistry of the culture media, material and surface area of the electrodes, as well as the ES regime, which influenced charge transfer during the stimulation (i.e. charge injection capacity).^16,76–78^

### MSC responses to direct ES and the roles of faradic by-products

ERK is one member of mitogen-activated protein kinases (MAPK) signalling cascade besides c-Jun N-terminal kinases (JNK) and p38.^79^ The activation of ERK1/2 signalling could lead to a variety of cellular responses, including MSC proliferation and differentiation.^79,80^ It was found in this study that direct ES has induced ERK1/2 phosphorylation within the first 30 min of stimulation, and the phosphorylation was prolonged for at least another 30 min of stimulation. The activation of ERK1/2 signalling in the electrically stimulated cells was consistent with an increase in c-FOS mRNA expression, which was the ERK1/2 transcription factor.^81^ Slight increase in c-JUN expression was also observed after 1 h of stimulation, which could also be induced by ERK1/2 signalling.^82^ Based on our current results, we were unable to confirm the involvement of JNK signalling. On the other hand, the upregulation of c-FOS and c-JUN mRNA expression was not significant when treating the cells with ES media for 1 h in comparison with cells treated with non-stimulated media. This finding implied that ERK1/2 phosphorylation and the transcription of c-FOS and c-JUN were induced by the electrical factors, not the faradic by-products. It could also be hypothesised that the signal from ES is transduced to the cells through MAPK pathways.^23^ Furthermore, it was found that the level of c-FOS and c-JUN mRNA expression were not significantly different from non-stimulated cells after 7 days of direct ES, which indicated that ERK1/2 signalling was only activated at the early stage of ES. In addition, the electrically induced ERK1/2 phosphorylation was also found when applying pulsed ES in both in vitro and in vivo.^83–85^

The expression of two osteogenic markers were characterised after 7 days of direct ES: SPP1; and RUNX2. SPP1 mRNA was responsible for translating osteopontin, which is a necessary protein for bone remodelling and mineralisation.^86,87^ Meanwhile, RUNX2 mRNA was expressed from the cells committed to the osteogenic lineage.^88^ It was found that direct ES has significantly increased the level of SPP1 mRNA expression, whilst having no effect on the expression of RUNX2 mRNA. This was consistent with the responses from pre-osteoblasts reported in our previous study that the ES regime used in this study does not directly promote cell differentiation towards the osteogenic lineage during the early stage, but influences their pro-osteogenic gene expression.^34^ Moreover, we have demonstrated that SPP1 mRNA expression could also be promoted by treating the cells with ES media, and the addition of sodium pyruvate did not significantly alter this upregulation. This finding suggested that the upregulation of SPP1 mRNA expression by ES media was not due to the electrically generated H_2_O_2_, but due to the other type of the faradic by-products. Whether or not this was also the case for macrophages in our previous study, this question would need to be addressed in future.^34^ Although the faradic by-products have not been fully characterised yet, we have analysed one candidate which was the dissolved Pt ion in the ES media. It has been reported that metallic ions have played a role in directing the activities of MSCs and osteoblasts.^89–91^ However, there were only distant correlations between Pt and MSC activities or osteopontin expression reported in the literatures.^92,93^ Otherwise, this response could also be induced by other products from any molecules that underwent redox reactions during direct ES.

Cell metabolic activity was also increased after 10 days of 1-h daily ES, which could be correlated with an increase in cell proliferation.^94^ Similar to SPP1 mRNA expression, it was found that cell metabolic activity could be increased by treating cells with ES media. However, changes in cell metabolic activity by ES media was not significant with the presence of sodium pyruvate, which suggested that the electrically generated H_2_O_2_ had a pivotal role in promoting MSCs proliferation. The enhancement of MSCs and other mammalian cell proliferation by exogenous H_2_O_2_ has been studied previously and it was suggested that physiological concentration of exogenous H_2_O_2_ was capable of promoting cell proliferation without causing oxidative damage.^95–97^ Although the mechanism was not well understood, one of the plausible mechanism could be the phosphorylation of the H_2_O_2_-responsive signalling proteins, that is, heterogeneous nuclear ribonucleoprotein C1/C2 (hnRNP-C1/C2), which consequently initiated cellular signalling towards cell proliferation.^97^ Furthermore, it was observed from this study that direct ES could also potentially be used as a tool to deliver the controllable amount of exogenous H_2_O_2_ to the cells.

It could be understood that an increase in SPP1 mRNA expression and proliferation of MSCs induced by ES were potentially through ERK1/2 and c-FOS and c-JUN transcription pathways, whereas the mechanisms involving faradic by-products may need further investigation.^98,99^ Consistent cellular responses to direct ES have been found in osteogenic media as well in terms of their SPP1 mRNA expression and metabolic activity. Strikingly, we also observed a significant decrease in ALP activity of the stimulated cells cultured in osteogenic media compared with the non-stimulated cells. It is important to point out the variation of cellular responses to direct ES as the previous studies suggest that MSCs derived from rat adipose or bone tissues have expressed osteogenic markers and undergone mineralisation, despite being stimulated with similar regime to this study.^38,39,49,100^ Nonetheless, we have noticed that in fact there was a difference in O_2_ level in the culturing condition as the direct ES system in the aforementioned studies with rat MSCs were incorporated with hypoxic condition (3–5% O_2_). Hence, it is plausible that the cellular responses to direct ES could also be influenced by the changes in O_2_ level.^101–103^ Otherwise, the reduction in ALP activity could also be due to the trade-off between cell proliferation and differentiation, since the stimulated cells in this experiment were more proliferative than non-stimulated cells.^104,105^ On the other hand, the reduction in ALP activity and mineralisation after ES was also found in rat dental pulp stem cells as reported by a recent study.^106^

### Clinical relevance of the findings from this study, limitations and future works

The findings from this study suggested that the increased MSC proliferation and the osteopontin expression from the MSCs could be involved in the mechanism of electrically induced osteogenesis found in the early studies. Our computational data also supported the ideas and hypotheses that the mechanism of ES could involve the changes in current density and extracellular potential, which regulated the level of intracellular Ca^2+^ and other ions by mediating the voltage-sensitive receptors and hyperpolarisation, respectively.^14,100,107,108^ These changes would then play a role in directing cellular activities and influence the MSC responses observed in this study.^100,109,110^ Although the influence of direct ES on the osteogenic differentiation of MSCs was not evident, it is expected that the electrically induced osteopontin expression from the MSCs would subsequently regulate bone remodelling and mineralisation.^86,87,110–112^ Furthermore, the role of electrically generated H_2_O_2_ and other faradic by-products in promoting proliferation and osteopontin expression from the MSCs were also observed, which could be another parallel mechanism. It is plausible that the electrically generated H_2_O_2_ could influence cells such as MSCs and osteoclasts nearby the injury site to promote remodelling of the defected bone tissue.^57,60,96,113,114^ In addition, the osteopontin expression from the electrically stimulated MSCs was consistent with the result observed previously in pre-osteoblasts and macrophages.^34^ Therefore, it could be thought that there were more than one cell type working synergistically in response to ES towards the osteogenesis, in vivo. However, it could still be asked whether or not the dissolved Pt was relevant when comparing with the in vivo and clinical studies as it was likely to be the product from the oxidation at the anode, which was usually implanted into the surrounding muscles and not at the injured bone tissue.^11,73^ This could be confirmed in future by comparing the cellular responses to anodic and cathodic ES media.

Moreover, the investigation of cellular responses to ES and bone matrix quantification after 28 days would also be worth investigating as it would strengthen the findings in terms of the long-term clinical outcome, as well as the investigation of the responses from the MSCs of different donors and/or different passages. On the other hand, incorporating ES with the biocompatible conductive scaffolds would also be another potential strategy for orthopaedic treatments as it could enhance cellular activities further towards bone regeneration.^115,116^

## Conclusion

The responses of MSCs to direct ES could be regulated by both non-faradic and faradic charges, which altered the physical and chemical conditions of the extracellular microenvironment and cell membrane. There were two hypothetical mechanisms concluded from this study. The first mechanism was the activation of ERK1/2 signalling and c-FOS and c-JUN transcription by the changes in electrolyte potentials and current density, which correlated with the increased SPP1 mRNA expression and cell metabolic activity. The second mechanism was induced by the faradic by-products in the ES media, including H_2_O_2_, which also resulted in the increases in SPP1 mRNA expression and cell metabolic activity. These mechanisms are believed to be associated with the electrically induced osteogenesis. We believe that these findings will be helpful for the development of scalable electrical stimulation systems for controlling the cell fate in future.

## Supplemental Material

sj-pdf-1-tej-10.1177_2041731420974147 – Supplemental material for Changes in the extracellular microenvironment and osteogenic responses of mesenchymal stem/stromal cells induced by in vitro direct electrical stimulation Click here for additional data file. Supplemental material, sj-pdf-1-tej-10.1177_2041731420974147 for Changes in the extracellular microenvironment and osteogenic responses of mesenchymal stem/stromal cells induced by in vitro direct electrical stimulation by Kasama Srirussamee, Ruikang Xue, Sahba Mobini, Nigel J Cassidy and Sarah H Cartmell in Journal of Tissue Engineering
