# Supplementary material for: Changes in the extracellular microenvironment and osteogenic responses of mesenchymal stem/stromal cells induced by in vitro direct electrical stimulation
Source: J Tissue Eng. 2021 Feb 16;12:2041731420974147. doi: 10.1177/2041731420974147 (PMC7894594; doi:10.1177/2041731420974147)
Supplement: sj-pdf-1-tej-10.1177_2041731420974147 – Supplemental material for Changes in the extracellular microenvironment and osteogenic responses of mesenchymal stem/stromal cells induced by in vitro direct electrical stimulation [file sj-pdf-1-tej-10.1177_2041731420974147.pdf]

Supplementary Materials and Methods

Calculation of empirical equilibrium potentials (E_{eq})

Following the total current measurement, the results were divided into 3 ranges based on the relationship between the total current and applied voltage: undetectable current; non-linear current; and linear current. Within the “undetectable current” region, it was assumed that there was no activation overpotential when applying voltage (E_{cell}) below 1.5 V, resulting in $E_{eq} = E_{cell}/2$. Within the second and third regions, the arbitrary ratio of $2E_{eq}/E_{cell}$, denoted by “x”, at each applied voltage were approximated from the measured total current by using model parametric sweeping as shown in figure S1(a, b). The value of “1 − x” at each region were then plotted against applied voltage, in which the empirical coefficient for E_{eq} in function of E_{cell} was obtained by fitting the plotted data points, as shown in figure S1(c, d). The computed total current using the obtained empirical coefficients were plotted in comparison with the measured total current in figure S1(e) to confirm the consistency of the input parameters. It is noted the empirical coefficients are dimensionless.

Supplementary Figures

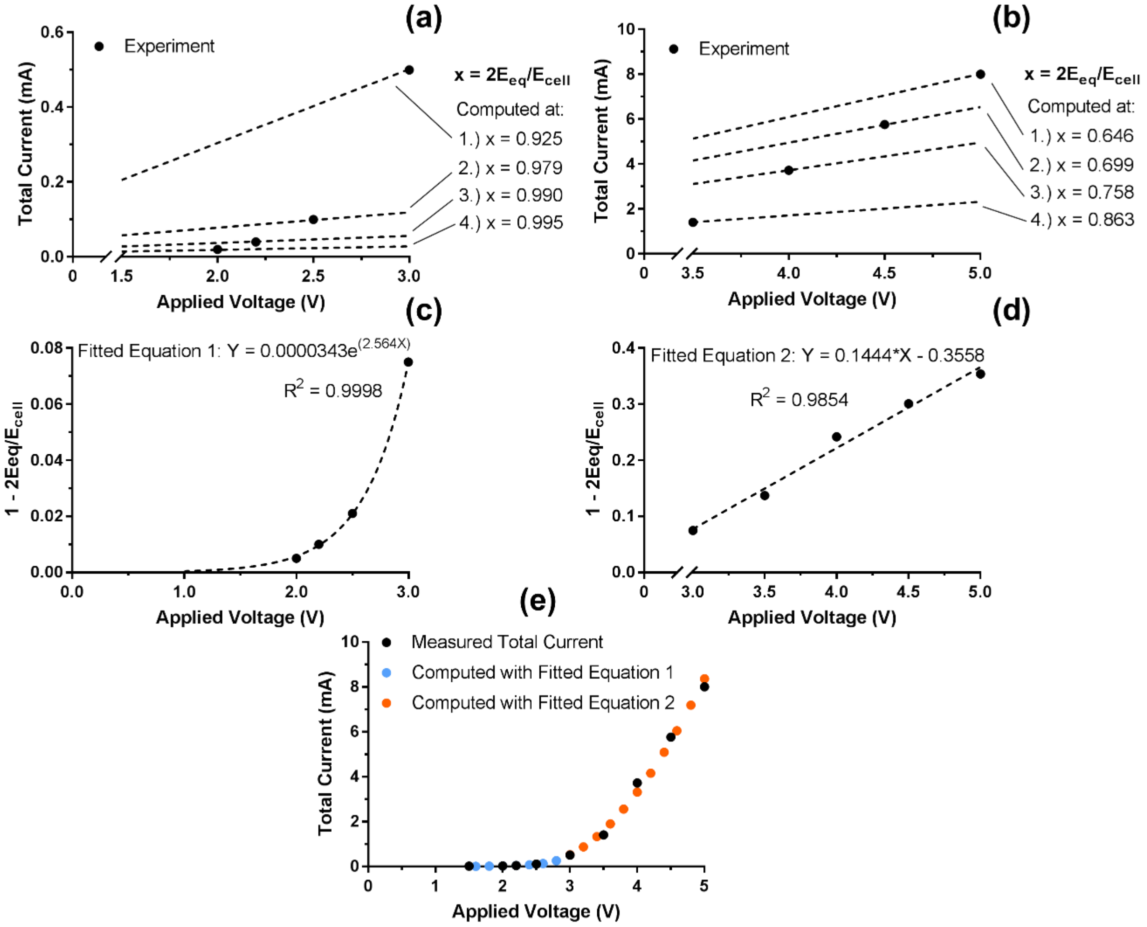

Figure S1 (a, b) Approximation of the arbitrary ratio "x" within the non-linear and linear current regions, respectively. (c, d) Fitted empirical equations for "1 – x" in function of the applied voltage (E_{cell}) within the non-linear and linear current regions, respectively. (e) Computed total current using the empirical coefficient from the fitted equations in comparison with the measured total current.

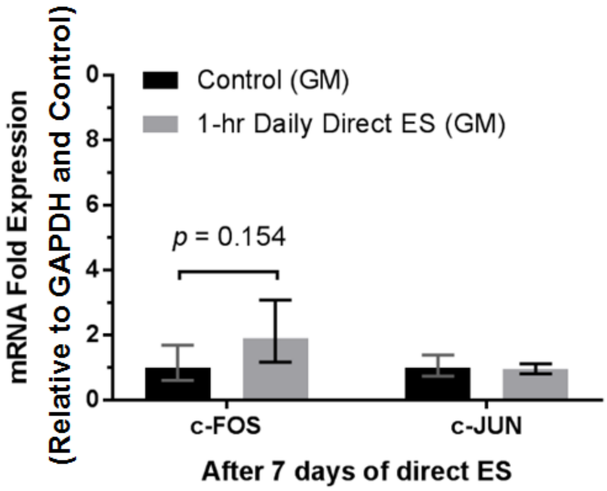

Figure S2 c-FOS and c-JUN mRNA expressions after 7 days of 1-hour daily direct ES in growth media (GM). Error bars represent upper and lower 95% confidence limits (n = 3 experiments). Statistical analyses were carried out using unpaired two-tailed Student’s t-test.
